# Disease-causing mutations in the *CLRN1* gene alter normal CLRN1 protein trafficking to the plasma membrane

**Published:** 2009-09-08

**Authors:** Juha Isosomppi, Hanna Västinsalo, Scott F. Geller, Elise Heon, John G. Flannery, Eeva-Marja Sankila

**Affiliations:** 1 Folkhälsan Institute of Genetics, Department of Molecular Genetics, Helsinki, Finland; 2 Department of Medical Genetics, University of Helsinki, Helsinki, Finland; 3 Helen Wills Neuroscience Institute, University of California, Berkeley, CA; 4 The Hospital for Sick Children, Department of Ophthalmology and Vision Sciences, Toronto, Ontario, Canada; 5 Helsinki University Eye Hospital, Helsinki, Finland

## Abstract

**Purpose:**

Mutations of clarin 1 (*CLRN1*) cause Usher syndrome type 3 (USH3). To determine the effects of USH3 mutations on CLRN1 function, we examined the cellular distribution and stability of both normal and mutant CLRN1 in vitro. We also searched for novel disease-causing mutations in a cohort of 59 unrelated Canadian and Finnish USH patients.

**Methods:**

Mutation screening was performed by DNA sequencing. For the functional studies, wild-type (WT) and mutant *CLRN1* genes were expressed as hemagglutinin (HA) tagged fusion proteins by transient transfection of BHK-21 cells. Subcellular localization of CLRN1-HA was examined by confocal microscopy. The N-glycosylation status of CLRN1 was studied by using the N-glycosidase F (PNGase F) enzyme and western blotting. Cycloheximide treatment was used to assess the stability of CLRN1 protein.

**Results:**

We found three previously reported pathogenic mutations, p.A123D, p.N48K, and p.Y176X, and a novel sequence variant, p.L54P, from the studied USH patients. The WT HA-tagged CLRN1 was correctly trafficked to the plasma membrane, whereas mutant CLRN1-HA proteins were mislocalized and retained in the endoplasmic reticulum. PNGase F treatment of CLRN1-HA resulted in an electrophoretic mobility shift consistent with sugar residue cleavage in WT and in all CLRN1 mutants except in p.N48K mutated CLRN1, in which the mutation abolishes the glycosylation site. Inhibition of protein expression with cycloheximide indicated that WT CLRN1-HA remained stable. In contrast, the CLRN1 mutants showed reduced stability.

**Conclusions:**

WT CLRN1 is a glycoprotein localized to the plasma membrane in transfected BHK-21 cells. Mutant CLRN1 proteins are mislocalized. We suggest that part of the pathogenesis of USH3 may be associated with defective intracellular trafficking as well as decreased stability of mutant CLRN1 proteins.

## Introduction

Usher syndrome (USH) describes a group of autosomal recessive diseases with bilateral sensorineural hearing loss and visual impairment phenotypically similar to retinitis pigmentosa (RP) [1-4]. Prevalence of USH in different populations is estimated to range from 3.5 to 6.2 per 100,000, thus making it the most frequent cause of combined deaf-blindness worldwide [5]. The condition has been classified into three clinical subtypes (USH1, USH2, and USH3), based on the severity and progression of the hearing impairment, presence or absence of vestibular dysfunction, and the age of onset of RP [1]. This classification remains in clinical use, although recent progress on the molecular genetics and clinical research of USH has revealed broad genetic and clinical heterogeneity [3,6]. Atypical forms of USH have been identified within all three clinical types, and there is considerable overlap of symptoms among the subtypes. A distinguishing feature of USH3 is the wide spectrum of nonlinear progressive hearing impairment, which ranges from a near normal to a severe audiometric phenotype [7]. USH3 patients may also have either normal or decreased vestibular responses [8]. The rate of visual loss in USH3 is similar to other USH subtypes [9], with the most recent analyses suggesting that retinal degeneration in USH3 progresses more rapidly than in USH2A [10,11]. The variable phenotype may cause USH3 to be under-diagnosed and it may be more prevalent than previously indicated [6].

To date, nine USH gene products have been identified: the molecular motor myosin VIIa (USH1B) [12]; the cell adhesion proteins cadherin 23 (USH1D) [13] and protocadherin 15 (USH1F) [14,15]; the scaffold proteins harmonin (USH1C) [16], SANS (USH1G) [17], and whirlin (USH2D) [18]; the G-protein-coupled 7-transmembrane receptor VLGR1b (USH2C) [19]; two isoforms of the extracellular matrix connected protein usherin (USH2A) [20,21]; and the four-pass transmembrane domain protein clarin 1 (USH3) [22,23]. There is growing evidence suggesting that these proteins form a network, which is critical for the development and maintenance of the sensorineural cells in the inner ear and the retina [3,4,24-28].

Since the original identification of the causative gene for USH3 [22], the gene’s structure has been refined. The newly defined *CLRN1* has three exons, encoding a 232 amino acid protein [23,29]. Northern blot and reverse-transcription PCR analyses indicate expression of different splice variants of *CLRN1* mRNA in several tissues including retina, cochlea, brain, and thymus [22,23,29]. In situ hybridization analyses demonstrate *Clrn1* expression in mouse cochlear hair cells and spiral ganglion cells as early as embryonic day (E) 16.5 [23,30]. The CLRN1 protein is thought to be expressed in mouse cochlea transiently from E18 to postnatal day (P) 6 in basal parts of the hair cells, whereas in apical parts (stereocilia) the CLRN1 expression is lost already at P1. In adult mouse retina CLRN1 localizes to inner segments, connecting cilia and ribbon synapses. The function of CLRN1 remains unknown; however, the spatiotemporal expression pattern of CLRN1 in hair cells implicates protein involvement in synaptic maturation [31]. Structural and sequence homology with the synaptic protein stargazin suggest a role for CLRN1 in the plasma membranes surrounding ribbon synapses of the inner ear and retina [23]. In cell culture studies CLRN1 forms microdomains in the plasma membrane, affects F-actin organization and induces lamellipodia formation, implicating CLRN1 involvement in actin cytoskeleton regulation [28]. This hypothesis is supported by the observation that *Clrn1* knockout mouse F-actin-rich hair cell stereocilia are disorganized [28,30]. In mouse hair cell cultures, however, CLRN1 associates with tubulin and not with actin, and localizes to post-transgolgi vesicles, suggesting a potential function for CLRN1 in vesicle transport [31].

To date, USH3 has been described as the rarest of the USH types worldwide. The availability of molecular diagnosis is increasing the number of USH3 cases identified. Interestingly, in unrelated Ashkenazi Jewish and Finnish populations, USH3 comprises 40% of all the USH cases, suggesting multiple founder effects [9,32]. Among Ashkenazi Jews of European and North American descent, a c.144T>G mutation is causative of the majority of the USH3 cases. This mutation causes a substitution of asparagine to lysine (p.N48K), and removes the single N-glycosylation consensus site on the CLRN1 protein [29]. The most common mutation in Finland is c.528T>G, which is predicted to generate a premature termination of the CLRN1 polypeptide (p.Y176X). Eleven other CLRN1 mutations have been documented in USH3 patients around the world. These mutations include c.359T>A (p.M120K) found in four Finnish patients, c.368C>A (p.A123D) found in one French Canadian patient, c.449T>C (p.L150P) found in one Ashkenazi Jewish patient, and c.459_461del (p.I153_L154delinsM) identified in two Italian patients. The other seven known CLRN1 mutations have been found in either single patients or isolated families with predominantly European ancestry [8,11,18,22,23,29,32-34].

In this study, we sequenced the coding region of *CLRN1* in 59 USH patients who had no known disease-causing mutations. Furthermore, we examined intracellular targeting, stability, and N-glycosylation of the aforementioned mutants in addition to WT CLRN1 using a transient transfection assay in BHK-21 cells.

## Methods

### Subjects

Patients diagnosed with USH were recruited through the Ocular Genetics Clinic at The Hospital for Sick Children in Toronto, Ontario, Canada and from Helsinki University Eye Hospital, Helsinki, Finland. This study followed the tenets of the Declaration of Helsinki.

### Clinical examinations

The diagnosis of USH was based on comprehensive ophthalmological and audiological examinations including Snellen visual acuity, biomicroscopy and fundus examinations, Goldmann visual field tests, electroretinograms, and pure tone audiograms. When possible, optical coherent tomography (OCT) was performed.

### Mutation analysis

DNA samples of 40 unrelated Canadian USH patients without previously known mutations and 19 USH patients diagnosed in Finland after 2001 were sequenced for *CLRN1* mutation identification. Patients’ genomic DNA was extracted either from fresh or frozen whole blood samples with Puregene™ Genomic DNA Purification Kit (Gentra Systems, MN) or from saliva with Oragene™ kits (DNA Genotek Inc., Ontario, Canada). A total of 90 Centre d’Etude du Polymorphisme Humain (CEPH) samples were used as controls for assessing the pathogenicity of the novel change found in this study from a Canadian USH patient. The three exons and the exon-intron boundaries of the main splice variant of *CLRN1* gene (GenBank accession NM_174878) were screened for mutations by genomic sequencing. The primers used for sequencing were: exon 0 sense 5′-CAG AAA AGG AGA AAA GCC AAG-3′; exon 0 antisense 5′-CTG GGA AGA GTC TGC CTA AA-3′; exon 2 sense 5′-TCA GAA GGA TTT TAG TGA TGT TTG A-3′; exon 2 antisense 5′-TCT TTT TGA CAT ATT GAA AAG CAC A-3′; exon 3 sense 5′-CCC TCT TCC CTG TCC CTT AC –3′; exon 3 antisense 5′- CCA CAT CTA AAA GTG ACC AAA GC-3′. The amplification conditions were 95 °C for 5 min (denaturation), then 30 cycles of 95 °C for 45 s, 60 °C (exon 2) or 55 °C (exons 0 and 3) for 45 s, 72 °C for 45 s, followed by a final extension of 10 min at 72 °C. PCR products were purified with Exo-SAP (USB, Cleveland, OH) and then sequenced with an ABI2720 Automatic DNA sequencer using the ABI PRISM BigDye® Terminator v3.1 Cycle Sequencing Kit (Applied Biosystems, Foster City, CA). The sequences were compared to the known *CLRN1* main splice variant sequence (NM_174878).

### Construction of expression plasmids

Wild-type (WT) *CLRN1* construct was cloned into the phCMV3 Xi cloning vector (Gene Therapy Systems, Inc., San Diego, CA) with C-terminal hemagglutinin epitope YPYDVPDYA (HA-tag) from the influenza virus A as recently described [28]. The mutant cDNA constructs representing mutations/sequence alterations p.N48K, p.L54P, p.M120K, p.A123D, p.L150P, and p.I153_L154delinsM were generated by the QuikChange site-directed in vitro mutagenesis kit, according to the manufacturer’s protocols (Stratagene, La Jolla, CA). All coding regions of the constructs were sequence verified.

### Cell culture and transfections

BHK-21 cells (CCL-10, ATCC) were cultured in Glasgow minimal essential medium (GMEM, Sigma-Aldrich, St. Louis, MO) supplemented with 10% fetal calf serum (FCS; PromoCell™, Heidelberg, Germany), Gibco™ GlutaMAX™ supplement (100x, L-alanyl-L-glutamine, Invitrogen), 5% tryptose phosphate broth solution (Sigma-Aldrich), and penicillin-streptomycin (100x, Sigma-Aldrich, Germany). For transfection, cells were seeded on 13 mm coverslips (Menzel-Gläser, Braunschweig, Germany) in six well plates (Nunc™, Denmark) at a density of 2×10^5^ cells per well 20 h before transfection. Transfection was performed with the FuGENE 6 Transfection Reagent (Roche Diagnostics, Indianapolis, IN) following the guidelines supplied by the manufacturer. Experiments were performed 24 or 48 h post transfection.

### Immunofluorescence imaging

WT and mutant CLRN1-HA transfected cells were fixed with 4% paraformaldehyde (PFA; pH 7.5) for 15 min before immunofluorescence staining. For stability analyses, protein synthesis was stopped by adding 50 µg/ml cycloheximide (Sigma-Aldrich) and incubating BHK-21 cells in it before fixation (as above). After fixation, BHK-21 cells were permeabilized for 20 min with 0.2% saponin (Sigma-Aldrich) in phosphate buffered saline (PBS; 137 mM NaCl, 2.7 mM KCl, 10 mM Na_2_HPO_4_, and 1.5 mM KH_2_PO_4_ in distilled water, pH 7.4) containing 0.5% bovine serum albumin (BSA; Sigma-Aldrich). Thereafter, the cells were incubated for 45 min with a 1:700 dilution of monoclonal anti-HA antibody (HA.11, MMS-101R; Covance, Berkeley, CA) and either with a 1:50 dilution of plasma membrane antibody to sodium potassium ATPase (ab7671; Abcam, Cambridge, UK), or a 1:200 dilution of an ER antibody to protein disulfide isomerase (PDI, spa-891; Stressgen, Victoria, Canada). Cy2- and Cy3–conjugated secondary antibodies (Jackson ImmunoResearch, West Grove, PA) were used to visualize the primary antibodies. The cells were mounted with Gel/Mount (Biomeda, Foster City, CA) and analyzed with a Leica DMR confocal microscope with TCS NT software, or a Zeiss Axioplan 2 microscope with AxioVision 3.1 software.

### Western blot and deglycosylation analyses

Transfected BHK-21 cells were harvested from six-well plates for western blot analysis by scraping in ice-cold PBS supplemented with protease inhibitors (Complete; Roche Diagnostics). Cells were collected by centrifugation. These were lysed with 200 µl glycoprotein denaturing buffer (0.5% SDS, 40 mM DTT; New England Biolabs, Ipswich, MA). Cells were further homogenized by passage through a 21 gauge needle. Next, 2 µl of 10% NP-40 and 2 µl of 50 mM sodium phosphate buffer, pH 7.5 (G7 Reaction Buffer) was added to the 15 µl sample of the cell homogenate. Samples for deglycosylation studies were treated with 0.5 µl (500 units/µl) of N-glycosidase F (PNGase F; New England Biolabs) enzyme for 2 h at 37 °C. Samples were separated on a 12% SDS–PAGE gel and transferred to a Trans-Blot® nitrocellulose membrane (Bio-Rad, Hercules, CA). The membrane was immunostained with a 1:1,000 dilution of monoclonal anti-HA antibody (Covance) and a 1:1,000 dilution of polyclonal rabbit anti-mouse IgG conjugated to HRP (DakoCytomation, Glostrup, Denmark). Bands were visualized by SuperSignal® West Pico Chemiluminescent Substrate (Pierce, Rockford, IL) and captured on X-ray film (Kodak Biomax MR Film, Sigma-Aldrich).

### Bioinformatics

The theoretical molecular weights of CLRN1-HA polypeptides were calculated with the Protein Molecular Weight Program [35]. The TMHMM2.0 program was used for prediction of membrane spanning regions [36].

## Results

### Sequence alterations in *CLRN1*

**Figure 1:**
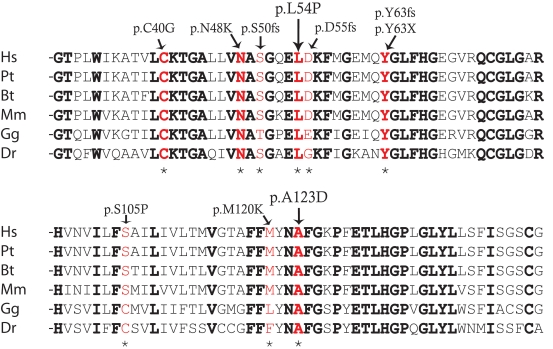
Sequence conservation around the p.L54P and p.A123D changes. Amino acids conserved in evolution are marked in bold, and mutations are marked with red. Mutations are also marked with an asterisk. Abbreviations: *Homo sapiens* (HS), *Pan troglodytes* (Pt), *Bos taurus* (Bt), *Mus musculus* (Mm), *Gallus gallus* (Gg), *Danio rerio* (Dr).

**Figure 2:**
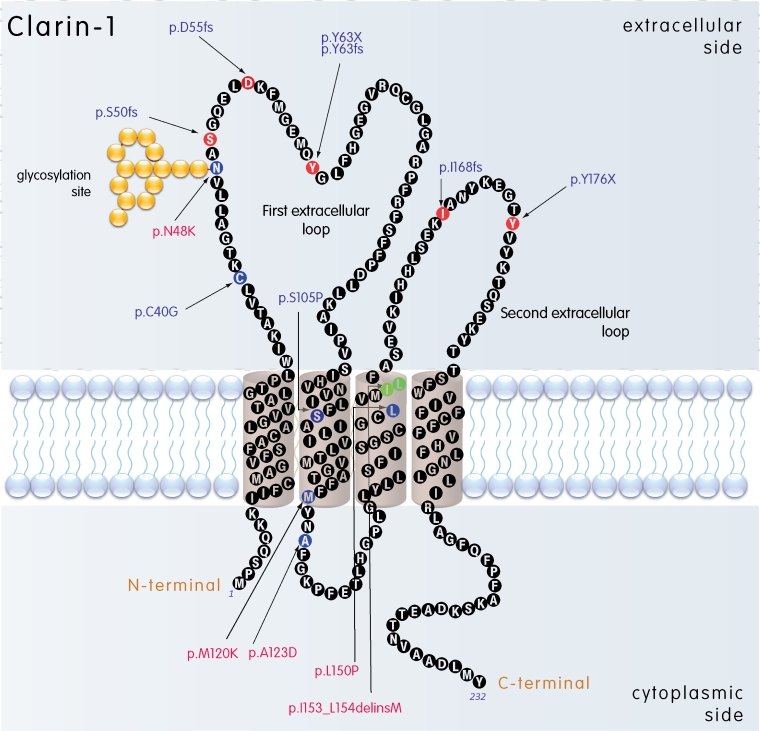
Predicted membrane topology of CLRN1. Disease-associated mutations are marked; the frameshift and nonsense mutations are marked with red amino acids, missense mutations are marked with blue amino acids, and the deleted amino acids replaced by an insertion are marked with green. Transmembrane regions (1–4) were predicted using a TMHMM2.0 program [36]. The mutations studied in this article are marked with red.

The sequencing of the *CLRN1* coding region from the 40 Canadian USH patients revealed two previously published and one novel *CLRN1* sequence alteration. The previously unreported c.161T>C transition, found in heterozygous form, predicts a p.L54P change. We did not find another *CLRN1* sequence alteration in this patient, but the changed amino acid leucine is conserved in evolution (Figure 1). Thus, the pathogenic nature of this alteration was investigated further. A transition mutation c.368C>A predicting a p.A123D change was identified in homozygous form in a Canadian patient originating from Dominica. The mutation changes another highly conserved amino acid alanine to aspartic acid (Figure 1). This change has previously been reported in a French Canadian patient [34] and is very likely a mutation causing USH3. Neither c.161T>C nor c.368C>A were found from the 90 CEPH control samples nor the 19 Finnish patients we sequenced. The Ashkenazi Jewish mutation (p.N48K) was detected in a Canadian USH patient in homozygous form, and in a Finnish patient in heterozygous form with the Finnish founder mutation p.Y176X. The mutations identified in the present study, as well as *CLRN1* disease-causing mutations identified in patients in other studies (Table 1), are summarized on a schematic presentation of the predicted *CLRN1* structure in Figure 2.

**Table 1:** CLRN1 mutations and their prevalences.

**USH3 mutation**	**Patient ethnicity**	**Note**	**Number of novel patients/families reported**	**Reference**
**p.C40G** **c.118T>G**	Spanish	homozygote	1/1	[33]
**p.N48K** **c.144T>G**	Eastern European Jewish	5/6 pts homozygotes, 1/6 heterozygote, other allele not found	6/4	[23]
	Ashkenazi Jewish	homozygote	16/11	[32]
	Ashkenazi Jewish	homozygote heterozygote with p.L150P	5/5 1/1	[29]
	Jewish (USA)	homozygote	5/5	[8]
	Ashkenazi Jewish	homozygote heterozygote, other allele not found	7/6 2/2	[11]
	Canadian	homozygote	1/1	This study
	Finnish	compound heterozygote with p.Y176X	1/1	This study
**p.S50fs c.149_152delinsTGTCCAAT**	Scotch-Irish (USA)	homozygote compound heterozygote with p.Y176X	1/1 1/1	[29]
	UK (USA)	homozygote	1/1	[8]
	German	compound heterozygote with c.502_503insA	3/1	[18]
**p.D55fs** **c.165delC**	Dutch (USA)	heterozygote, other allele not found	1/1	[29]
	Dutch (USA)	heterozygote, other allele not found	4/1	[8]
**p.Y63fs** **c.187_209del**	Yemenite Jewish	homozygote	2/1	[23]
**p.Y63X** **c.189C>A**	Spanish	homozygote	3/1	[23]
**p.S105P** **c.313T>C**	Turkish	homozygote	2/1	[8]
**p.M120K** **c.359T>A**	Finnish	compound heterozygote with p.Y176X	4/2	[22]
**p.A123D** **c.368C>A**	French Canadian	homozygote	1/1	[18]
	Dominican (Canadian)	homozygote	1/1	This study
**p.L150P** **c.449T>C**	Ashkenazi Jewish	compound heterozygote with p.N48K	1/1	[29]
**p.I153_L154delinsM c.459_461del**	Italian	homozygote	4/1	[22]
**p.I168fs** **c.502_503insA**	German	compound heterozygote with c.149_152delinsTGTCCAAT	3/1	[18]
**p.Y176X** **c.528T>G**	Finnish	homozygote	52/21	[22]
	Northern European, one family Scotch-Irish (USA)	homozygote	11/6	[29]
	Finnish-Swedish	homozygote	13/5	[8]
	Scotch-Irish (USA)	compound heterozygote with c.149–152del	3/1	[8]

Disease-causing USH3 mutations identified in patients are listed with the number of patients and families reported to be affected by these mutations.

### Clinical findings in the patients whose mutations were found in this study

**Figure 3:**
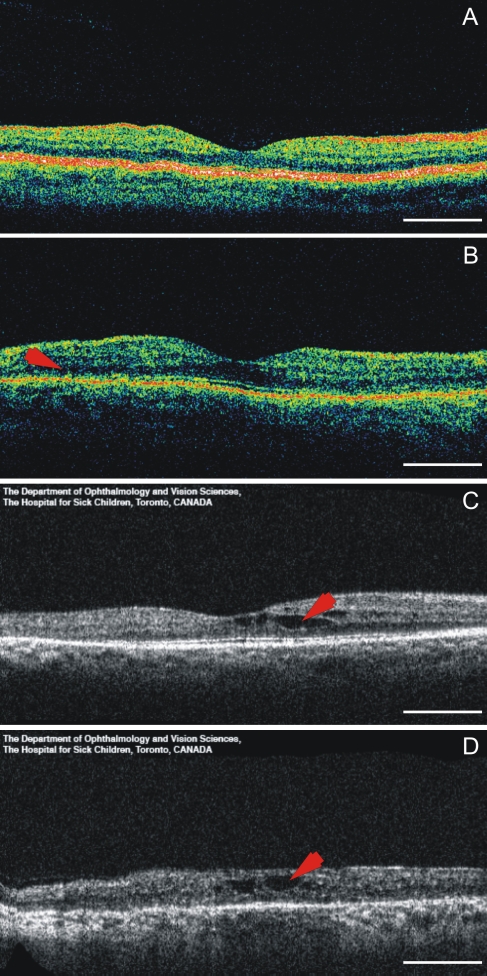
Optical coherence tomographs of a normal control and three USH3 patients. **A**: 32-year-old healthy control with visual acuity (VA) of 20/20. **B**: 8-year-old USH3 patient with heterozygous p.Y176X and p.N48K mutations and VA of 20/20. The arrow points to the region of retinal thinning in patient’s macula. **C**: 35-year-old USH3 patient with homozygous p.A123D mutation and VA of 20/25. The arrow points to the schisis-like change in patient’s macula. **D**: 52-year-old USH3 patient with homozygous p.N48K mutation and VA of 20/30. The arrow points to the intraretinal cysts. Scale bar represents 1 mm.

The Canadian patient with a homozygous p.N48K mutation was diagnosed with RP and hearing loss in his late teens. At age 54, he had 5 degree visual fields with the III4e stimulus. His best-corrected visual acuities were 20/30 OU (following cataract surgery). He had a mild to moderate, downsloping sensorineural hearing loss and poor balance. OCT showed small cystic macular changes at age 52 (Figure 3D). The Canadian patient with the homozygous p.A123D mutation had progressive sensorineural hearing loss that was first noted at age 7. By age 32, her hearing loss was moderately severe, sloping to profound hearing loss. She noticed symptoms of RP at age 25, when her ERG was nonrecordable. At 36, her visual fields were 12 degrees measured with the III4e stimulus, and the best-corrected visual acuities were 20/25 OU. On OCT, there was a schisis-like change in her macula (Figure 3C). Fundus changes were otherwise characteristic of RP. The Finnish p.N48K/p.Y176X compound heterozygous patient was diagnosed with sensorineural hearing loss at age 7; testing revealed a U-shaped audiogram. At age 8, his visual acuity was 20/20 OU. He had slight mottling of the pigment in his peripheral fundi, and his visual fields, measured with the V4e standard light, were normal. His full-field ERGs were nonrecordable. On OCT examination, the foveal thickness looked fairly normal but there was thinning of the retina outside the central fovea (Figure 3B).

### Cellular localization of CLRN1-HA

**Figure 4:**
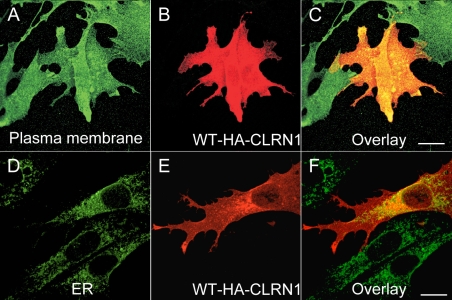
Cellular localization of WT CLRN1-HA protein in transfected BHK-21 cells. In panels **B** and **E** the cells were immunostained with HA antibody (red). In panel **A** the cells were immunostained with a plasma membrane specific antibody (green) and in panel **D** with ER specific antibody (green). The right-most panels (**C** and **F**) show the overlay of both CLRN1-HA and the organelle-specific double staining. Yellow-orange staining indicates an overlap of the CLRN1-HA protein (red) and subcellular markers (green). Cells were viewed with a confocal immunofluorescence microscope, magnification 63×. Scale bar represents 10 μm.

**Figure 5:**
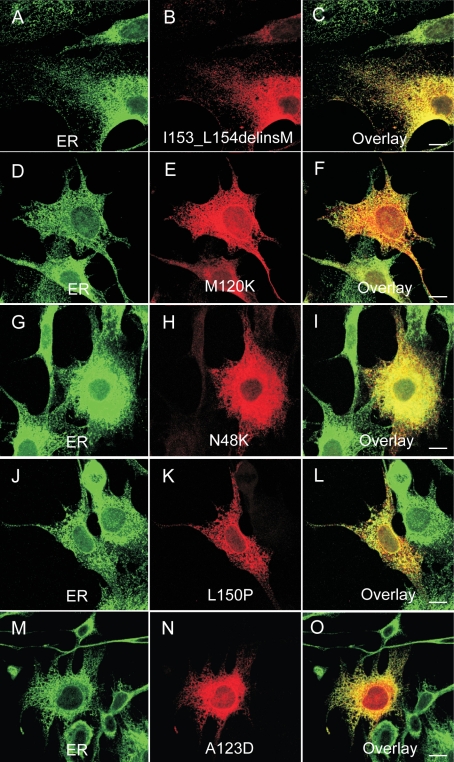
Cellular localization of the disease-causing mutant CLRN1-HA polypeptides. The transfected BHK-21 cells were double immunostained with HA antibody (red) in panels **B**, **E**, **H**, **K**, and **N** showing the localization of mutant CLRN1-HA. In panels **A**, **D**, **G**, **J**, and **M** the cells were stained with the ER marker (green). The right-most panels **C**, **F**, **I**, **L**, and **O** show the overlay of the mutant CLRN1- HA staining (red) and ER-specific staining (green). Yellow-orange staining indicates colocalization of these stainings. Cells were viewed with a confocal immunofluorescence microscope. Scale bar represents 10 µm.

**Figure 6:**
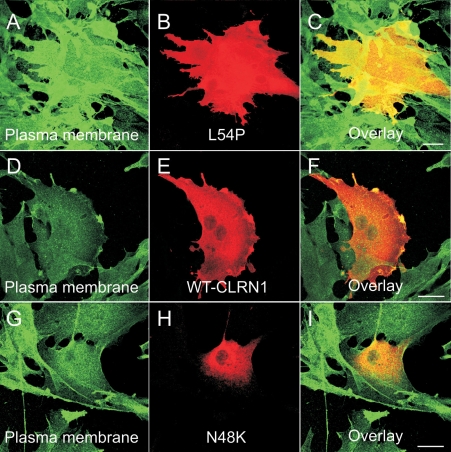
Cellular localization of the p.L54P, p.N48K and WT CLRN1-HA polypeptides. The transfected BHK-21 cells were immunostained with HA antibody (red) showing the localization of WT CLRN1-HA (**E**), the novel sequence alteration p.L54P mutated CLRN1-HA (**B**) and the known disease-causing p.N48K mutated CLRN1-HA (**H**). The same cells were immunostained with plasma membrane –specific antibody (green) in panels **A**, **D**, and **G**. Double-staining shows that WT CLRN1-HA (**F**) and the p.L54P mutated CLRN1-HA (**C**) colocalize (yellow) with the plasma membrane marker whereas the known mutation p.N48K (**I**) does not colocalize with the plasma membrane marker. Cells were viewed with a confocal immunofluorescence microscope. Scale bar represents 10 µm.

To determine the cellular localization of the CLRN1 protein, we cloned the transcript of *CLRN1* (NM_174878) into a HA-tagged phCMV mammalian expression vector. We generated mutant cDNA constructs by the QuikChange site-directed mutagenesis kit. The mutant and WT proteins were transiently expressed in BHK-21 cells. Their refined cellular localizations were visualized by anti-HA, anti-ER (protein disulfide isomerase), and anti-plasma membrane (sodium potassium ATPase) antibodies, using confocal immunofluorescence microscopy. WT CLRN1 was observed throughout the plasma membrane of transfected cells (Figure 4A-C), but it also showed partial colocalization with the ER marker (Figure 4D-F), indicating its normal processing through the ER to the plasma membrane. In contrast, all known mutant proteins analyzed colocalized almost exclusively within the ER, suggesting that they are retained therein and prevented from targeting to the plasma membrane (Figure 5). The novel sequence alteration p.L54P localized to the plasma membrane (Figure 6A-C) similar to WT CLRN1 (Figure 6D-F), and unlike the known mutation p.N48K (Figure 6G-I). The biologic significance of the p.L54P alteration found in a patient in heterozygous form remains thus unclear.

### Stability of WT and mutant CLRN1-HA polypeptides

**Figure 7:**
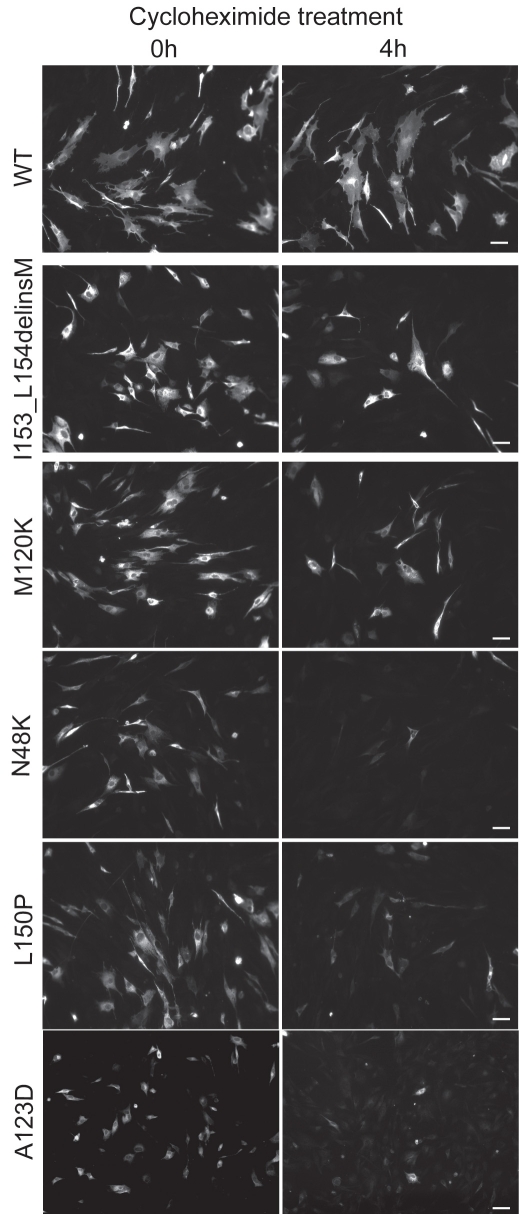
Stability of WT and mutant CLRN1-HA polypeptides in transfected BHK-21 cells. Cells were transiently transfected either with WT or mutant CLRN1-HA cDNAs. At 48 h posttransfection protein synthesis was stopped by incubating the cells for 4 h in the presence of 50 µg/ml of cycloheximide. Cells were viewed with a Zeiss Axioplan 2 fluorescence microscope. The scale bar represents 50 µm.

We studied the stability of the WT and mutant CLRN1-HA proteins by interrupting protein synthesis in transfected BHK-21 cells by cycloheximide treatment and examining how much protein remained after 4 h. WT CLRN1, the p.I153_L154delinsM and p.M120K mutants could still be detected, while the p.N48K, p.A123D and p.L150P mutants were almost completely absent (Figure 7). ER marker served as a control. Stability of the marker was unaltered by cycloheximide treatment (data not shown).

### CLRN1-HA forms multimers

**Figure 8:**
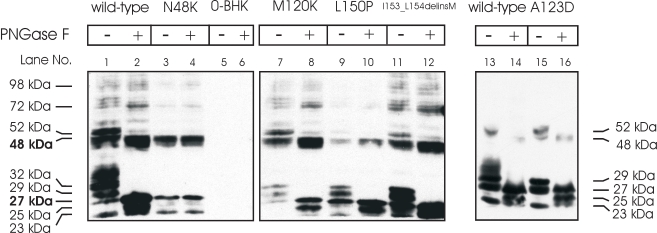
Western blot analysis of the wild-type and mutant CLRN1-HA polypeptides. BHK-21 cells were transfected with the indicated HA-tagged CLRN1 plasmids. Nontransfected cells (0-BHK) were used as controls. Polypeptides were resolved on 12% SDS–PAGE, and anti-HA antibodies were used to probe the blots. Samples were untreated (-) or treated (+) with deglycosylating enzyme (PNGase F). The molecular weights of the protein bands are indicated on the left and right sides of the figure.

Based on computational predictions, the molecular weight of WT CLRN1-HA with no modifications is 26.81 kDa [35]. WT CLRN1-HA has a single putative N-glycosylation site at amino acid position 48 [23,28]. To study CLRN1 glycosylation in vitro, we treated HA-tagged WT CLRN1 and p.N48K mutated CLRN1 polypeptides produced in transiently transfected BHK-21 cells with PNGase F, an enzyme that removes all N-linked oligosaccharide side chains from glycoproteins. Western blot analysis of WT CLRN1-HA showed several bands ranging from 23 up to 98 kDa (Figure 8, lane 1). After PNGase F treatment, two major bands at 27 kDa and 48 kDa were observed (Figure 8, lane 2). In contrast, PNGase F treatment had no effect on p.N48K samples: the same 23, 27, and 48 kDa bands were observed in both PNGase F treated and untreated samples (Figure 8, lanes 3 and 4). These results confirm that CLRN1 is a glycoprotein like previously reported and that the p.N48K mutation disrupts the protein’s single glycosylation site [28]. The presence of a 48 kDa band suggests that CLRN1 has a tendency to form dimers. The other studied mutations, p.M120K, p.A123D, p.L150P, and p.I153_L154delinsM, were glycosylated (Figure 8, lanes 7–16), and clearly showed decreased molecular weights following PNGase F treatment. Their glycoform patterns were, however, different from that of the WT, probably reflecting their retention in the ER.

## Discussion

A total of 13 *CLRN1* mutations in more than 100 USH3 patients have been reported worldwide. The two most common single mutations are the founder mutations p.Y176X in the Finnish population, and the p.N48K mutation among Ashkenazi Jews [22,23,32]. The remaining 11 reported *CLRN1* mutations occur in single, often consanguineous families, and are not known to be widely distributed. Therefore, it is not surprising that we found one p.N48K homozygote among 40 Canadian USH patients, and one compound p.Y176X/p.N48K heterozygote in Finland. The p.A123D mutation was found in a Canadian patient originating from Dominica. Interestingly, the same mutation has previously been reported in a French Canadian USH1 patient [34].

Clinical studies of USH3 patients have failed to determine any clear-cut genotype-phenotype correlations while broad intrafamilial and mutational variety in the onset, progression, and severity of symptoms are reported among the largest patient groups, Ashkenazi Jews [11,32], and Finns [7,9,10]. The homozygous p.A123D patient, the homozygous p.N48K patient, and the combined heterozygous p.Y176X/p.N48K patient reported in this study had what can be considered typical USH3 phenotypes.

The *CLRN1* gene is predicted to encode a four-pass transmembrane domain protein [23]. Our results are in line with the previous observations that full-length WT CLRN1 is sorted to specific membrane compartments or to the cell surface [28,31]. Interestingly, molecular masses of the CLRN1-HA polypeptides observed on our western blots suggest that CLRN1 may form dimers and even higher order complexes. The function of the protein still remains unclear. The eight other known USH gene products are hypothesized to form an USH interactome with a role in the molecular transport in the photoreceptor connecting cilium, and in the development and function of cochlear hair cells [3,26-28]. The possible role of CLRN1 in the USH interactome, however, remains unclear.

Half of the known *CLRN1* mutations result in a premature termination codon and are likely to lead to nonfunctional proteins. Alternatively, the mutant mRNAs may be degraded by the nonsense-mediated decay pathway [37]. To assess pathogenic mechanisms of nontruncating mutations, we studied consequences of four missense mutations (p.N48K, p.M120K, p.A123D, and p.L150P), and that of the p.I153_L154delinsM mutation by expressing HA-tagged mutant cDNAs in BHK-21 cells. We showed that all the mutant CLRN1 proteins studied are retained in the ER and not trafficked to the plasma membrane. This is in line with the fact that missorting and mistrafficking of membrane proteins is a well-described mechanism of cell degeneration [38,39].

We also studied the stability of WT and mutant proteins and found that all studied mutants were less stable than WT CLRN1-HA. The p.N48K, p.A123D and p.L150P mutants degraded more rapidly than p.M120K and p.I153_L154delinsM. A recent study with HEK-293 cells shows that disruption of glycosylation site by p.N48K mutation as well as removal of the N-linked sugar residue from the WT-CLRN1 both cause protein degradation, suggesting that the incorrect glycosylation is the cause for p.N48K mutated CLRN1 degradation [28]. Our study showed that mutations other than p.N48K are glycosylated, yet unstable. Therefore, it seems that glycosylation is not the sole determining factor for destabilization of mutated CLRN1 proteins in general. The accumulation of mutated proteins that cells inefficiently degrade may cause ER stress [40,41]. Retained as well as improperly processed mutant CLRN1 polypeptides are likely to cause ER stress, which in turn may trigger apoptosis in retinal and cochlear sensory epithelia. In a previous study, we showed that apoptosis is a probable pathogenic mechanism in USH3, as apoptotic cochlear cell destruction resulted from adeno-associated viral vectored ribozyme-initiated knockdown of *Clrn1* in the mouse cochlea [42].

In summary, our results showed that CLRN1 is a glycoprotein that forms multimers in plasma membranes of cultured cells. Further, we showed that instability and defective routing of mutant polypeptides are critical aspects of the etiology of USH3.
